# Severe Late-Onset Abiraterone-Induced Hypokalemia in a Diabetic Patient With Concomitant Adrenal Gland Incidentaloma: A Diagnostic Challenge

**DOI:** 10.1155/crie/8841993

**Published:** 2025-06-05

**Authors:** Andrea Tumminia, Francesco Galeano, Vittorio Oteri, Federica Gambero, Stefania Panebianco, Roberto Baratta, Daniela Leonardi, Ilenia Marturano, Dario Giuffrida, Francesco Frasca, Damiano Gullo

**Affiliations:** ^1^Endocrine Unit, Garibaldi-Nesima Hospital, Catania, Italy; ^2^Endocrinology Section, Garibaldi-Nesima Hospital, Department of Clinical and Experimental Medicine, University of Catania, Catania, Italy; ^3^Medical Oncology Unit, Mediterranean Institute of Oncology, Viagrande, Italy

**Keywords:** abiraterone, amiloride, dexamethasone, diabetes mellitus, hypokalemia, mineralocorticoid excess syndrome, prostate cancer

## Abstract

Prostate cancer is the most prevalent cancer among men in Western countries and is commonly managed by androgen deprivation therapy for locally advanced or metastatic stages. Even if initially effective, most patients eventually develop resistance to this treatment. Approved in 2011 for castration-resistant prostate cancer, abiraterone acetate inhibits the CYP17A1 enzyme, which is crucial in androgen and cortisol synthesis. This inhibition disrupts feedback on adrenocorticotropic hormone (ACTH), causing mineralocorticoid excess syndrome (MES), which is characterized by fluid retention, hypokalemia, and hypertension. MES can persist even with glucocorticoid supplementation, as observed in some cases. This study describes the case of a 68-year-old male with prostate cancer who developed severe, treatment-resistant hypokalemia after 6 years of abiraterone and prednisone therapy. The patient presented with poorly controlled diabetes and notable hypokalemia despite oral and parenteral potassium supplementation. Imaging revealed an adrenal adenoma; however, low renin and aldosterone levels suggested that abiraterone-induced MES, rather than primary aldosteronism, was responsible for his hypokalemia. The main therapy adjustment consisted of switching prednisone to dexamethasone to enhance ACTH suppression, effectively resolving the patient's hypokalemia. This case underscores the need for MES monitoring in patients on abiraterone, as MES can develop or worsen over time. Physicians should consider dexamethasone over prednisone in persistent MES cases, always monitoring also for the risk of developing Cushing syndrome. Given the rising prostate cancer incidence, clinicians must remain vigilant for MES-related complications with abiraterone, including delayed-onset severe hypokalemia.

## 1. Introduction

Prostate cancer is the most prevalent cancer in males in Western countries, and androgen deprivation therapy is the primary treatment for locally advanced and metastatic prostate cancer. While initially effective, most patients eventually develop castration-resistant prostate cancer, requiring alternative therapeutic strategies.

**Figure 1:**
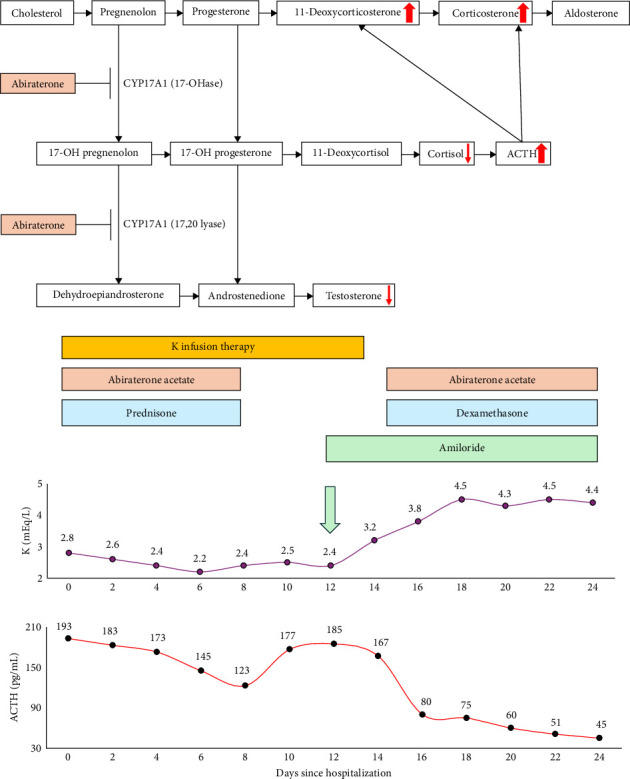
(A) Overview of the adrenal cortex steroid synthesis pathway. Abiraterone targets and inhibits the CYP17A1 enzyme, which is responsible for two key activities: 17-alpha hydroxylase and 17,20 lyase. By blocking these functions, abiraterone reduces cortisol production, resulting in decreased negative feedback on ACTH. Additionally, the drug suppresses androgen synthesis. The persistent ACTH stimulation promotes continued steroidogenesis, causing a buildup of precursor hormones before the CYP17A1 blockade, which in turn leads to increased mineralocorticoid production. The diagram illustrates the effects of CYP17A1 inhibition by abiraterone on the levels of mineralocorticoids, cortisol, and androgens, indicated by the red directional arrows. (B) Time-related serum potassium and ACTH changes before and after the introduction of amiloride and dexamethasone.

In 2011, the Food and Drug Administration authorized abiraterone acetate for treating castration-resistant prostate cancer. This oral agent offers a targeted approach, providing an alternative to traditional cytotoxic chemotherapy. Despite its proven efficacy and long-term safety, abiraterone is associated with significant adverse effects related to its mechanism of action [1]: specifically, by inhibiting key enzymes in the steroidogenesis pathway, abiraterone reduces the synthesis of both androgens and cortisol (Figure 1A).

However, the decrease in glucocorticoid production reduces the negative feedback on adrenocorticotropic hormone (ACTH). Elevated ACTH consequently stimulates the synthesis of corticosterone and deoxycorticosterone, leading to mineralocorticoid excess syndrome (MES), characterized by fluid retention, hypokalemia, metabolic alkalosis, and hypertension [2].

Supplementation with glucocorticoids (e.g., prednisone or dexamethasone) is recommended to reduce ACTH overproduction. Nonetheless, a portion of individuals treated with abiraterone continue to develop MES, which usually appears within 24 weeks of treatment [2].

We present the case of a prostate cancer patient who developed severe and refractory hypokalemia after 6 years of treatment with abiraterone.

## 2. Case Presentation

A 68-year-old man with a 10-year history of type 2 diabetes mellitus (T2DM) and high blood pressure was admitted to our Endocrinology Department in May 2024 for uncontrolled diabetes. He was diagnosed with prostate cancer in 2015 and underwent radical prostatectomy, followed by radiotherapy in 2016. Since 2018, he has been taking 500 mg of abiraterone and 5 mg of prednisone each morning.

At the time of admission, he was on metformin (1000 mg/day), had a blood glucose level of 340 mg/dL (reference: 70–100 mg/dL), and a glycated hemoglobin of 12.8% (reference: 4.0%–5.6%). The patient's body mass index was 32.2 kg/m^2^. His blood pressure was under good control using doxazosin 2 mg daily. On clinical examination, there were no signs of fluid overload, and a CT scan of the chest revealed no abnormalities associated with pulmonary edema or other manifestations of fluid retention, indicating that the patient did not have the entire clinical triad of MES. He had not taken diuretic medication in the past 6 months.

Laboratory testing revealed a severe hypokalemia, 2.2 mmol/L (reference: 3.5–5.1 mmol/L) and an increased 24-h urinary potassium excretion, 147 mmol/24 h (values >30 mmol/24 h indicating inappropriate renal potassium loss). Creatine phosphokinase was within normal limits, thus excluding rhabdomyolysis, rarely associated with abiraterone therapy [3], and a potential cause of hypokalemia.

Venous blood gas analysis showed a pH of 7.42 (reference: 7.31–7.41), bicarbonate 28.2 mmol/L (reference: 24–30 mmol/L), pCO_2_ 44.4 mmHg (reference: 40–50 mmHg), indicating a mild compensated metabolic alkalosis associated with the patient's hypokalemia.

The patient's glucose control improved after starting a hypocaloric diet, increasing the dose of metformin (from 1000 to 2000 mg daily), and adding liraglutide up to 1.8 mg/day without using insulin. At 3 months, his glycated hemoglobin was 7.2%, and by 6 months, it had dropped to 5.8%. However, serum potassium levels potassium levels did not change significantly over the following 6 days of hospitalization despite oral and parenteral potassium supplementation (150 mmol/day) (Figure 1B).

An abdomen CT scan showed a hypodense, 2-cm-sized nodular lesion in the left adrenal gland that had benign densitometric characteristics (<10 Hounsfield units). This finding, combined with the patient's potassium levels, raised concerns for primary aldosteronism.

A series of endocrine tests were done to investigate the adrenal adenoma further. The results revealed low levels of renin 0.3 pg/mL (reference: 2.7–16.5) and aldosterone 1.9 pg/mL (reference: 2.2–35.5 pg/mL). The serum cortisol level was 1.5 µg/dL (reference: 3.7–19.4), which, despite concurrent prednisone therapy, remained low, whereas ACTH levels were 193 pg/mL (reference: 10–60 pg/mL), suggesting that the prednisone dose was insufficient to adequately suppress ACTH and compensate for the cortisol deficiency induced by abiraterone. Testosterone was 0.04 ng/mL (reference: 2.7–9.6 ng/mL), and DHEA was 11 µg/dL (reference: 48–360 µg/dL). Urine catecholamine and metanephrine levels were within the normal range.

Although confirmatory testing for primary aldosteronism (e.g., saline-loading test) is generally recommended when Conn's syndrome is strongly suspected, in our patient, the combination of severe hypokalemia and markedly suppressed renin and aldosterone levels was incompatible with primary aldosteronism or renal artery stenosis. Furthermore, provocation testing was contraindicated by the risk of exacerbating electrolyte imbalance and fluid retention. We therefore considered these clinical and biochemical findings sufficient to exclude Conn's syndrome and to support a diagnosis of abiraterone-associated mineralocorticoid excess in this context [4].

To reduce potassium loss, we then started treatment with amiloride (5 mg/day), which selectively inhibits the renal epithelial mineralocorticoid-sensitive sodium channel. We opted not to use steroidal aldosterone receptor blockers (e.g., spironolactone and eplerenone), as these drugs can activate both wild-type and mutant androgen receptors, potentially aggravating castration resistance [2].

In our case, the recommended dose of prednisone in patients treated with abiraterone [2] resulted in inadequate ACTH suppression. Prednisone 5 mg in the morning was replaced with dexamethasone 0.5 mg in the evening. The duration of effect for prednisone is 18–36 h, and more than 36 h for dexamethasone, which we administered in the evening to achieve a more effective reduction in ACTH levels [5].

The patient's hypokalemia quickly resolved following these therapeutic modifications (Figure 1B). At discharge, the patient's renin levels had returned to a normal value of 2.4 pg/mL, and his ACTH level had dropped to 45 pg/mL.

## 3. Discussion

Abiraterone is a potent, selective, and irreversible inhibitor of cytochrome P-450c17 (CYP17A1), a key enzyme in the steroidogenesis pathway that plays a crucial role in the adrenal glands, testes, and prostate cancer cells [2]. CYP17A1 has two distinct functions: acting as a 17,20 lyase and as a 17-alpha hydroxylase. By inhibiting these enzymes, abiraterone reduces the synthesis of both androgens and cortisol (Figure 1A).

However, the resultant decrease in cortisol levels leads to a compensatory increase in ACTH due to the loss of negative feedback. This elevation in ACTH subsequently drives the accumulation of 11-deoxycorticosterone and corticosterone. Notably, corticosterone has an affinity for the mineralocorticoid receptor comparable to that of aldosterone, and its ~40-fold increase can induce a syndrome resembling mineralocorticoid excess. Clinically, this syndrome is, in most cases, characterized by fluid retention, hypokalemia, metabolic alkalosis, and hypertension. These adverse effects can be effectively mitigated by the simultaneous administration of glucocorticoids, such as prednisone or dexamethasone [1]. This is, to our knowledge, the first report of a late-onset (6 years) abiraterone-induced severe hypokalemia despite prednisone therapy. Several reports have shown the risk of an early onset of moderate-to-severe hypokalemia during the first few months of treatment with abiraterone [2]. Since the pathophysiology of abiraterone-induced MES is related to an elevation of ACTH due to enzymatic inhibitions, in some instances, like our patient's case, more effective and sustained ACTH suppression may be required [2]. Dexamethasone, with its higher glucocorticoid potency and lack of mineralocorticoid activity, offers a pharmacologic advantage over prednisone in this setting. Supporting this, a retrospective study showed a significantly higher prevalence of hypokalemia when abiraterone was coadministered with prednisolone (28.6%) compared to after switching to dexamethasone (7.1%) [6]. These findings suggest that dexamethasone may be more effective in controlling persistent MES. However, dexamethasone should be used cautiously to prevent iatrogenic Cushing syndrome.

A further peculiarity of this case is the concurrent detection of an adrenal incidentaloma, which may have led to the mistaken conclusion that his low serum potassium levels were caused by Conn's syndrome rather than abiraterone treatment.

Given the rising incidence of prostate cancer [7] and the established use of abiraterone in castration-resistant prostate cancer patients, physicians who are caring for these individuals must be cautious about the medication's possible endocrine adverse effects. Signs and symptoms of MES, particularly hypokalemia, should be monitored throughout the entire course of the treatment and not just in the early stages.

The use of dexamethasone instead of prednisone could be considered in some patients to obtain better suppression of ACTH levels, if MES does occur. The decision to switch glucocorticoids in the setting of abiraterone-induced MES should be guided by a combination of biochemical and clinical parameters. Persistent hypokalemia despite adequate potassium supplementation, low cortisol levels despite prednisone therapy, and elevated ACTH concentrations suggest insufficient glucocorticoid effect and warrant reassessment of the steroid regimen. In such cases, clinicians may consider switching to dexamethasone, particularly when ACTH remains elevated and hypokalemia is refractory. Monitoring ACTH and serum potassium levels provides a practical approach to evaluate treatment response and guide further adjustments.

## 4. Conclusion

This case underscores the need for MES monitoring in patients on abiraterone, as MES can develop or worsen over time. Physicians should consider dexamethasone over prednisone in persistent MES cases, always monitoring also for the risk of developing Cushing syndrome. Given the rising prostate cancer incidence, clinicians must remain vigilant for MES-related complications with abiraterone, including delayed-onset severe hypokalemia.

## Data Availability

Data sharing is not applicable to this article as no new data were created or analyzed in this study.

## Nomenclature

17*α*-OHase: 17*α*-Hydroxylase
CYP: Cytochrome p450 enzyme
ACTH: Adrenocorticotropic hormone.
